# Supplementary material for: Sorption and Desorption Studies of Pb(II) and Ni(II) from Aqueous Solutions by a New Composite Based on Alginate and Magadiite Materials
Source: Polymers (Basel). 2019 Feb 15;11(2):340. doi: 10.3390/polym11020340 (PMC6419164; doi:10.3390/polym11020340)
Supplement: Supplementary file 1 [file polymers-11-00340-s001.pdf]

Supplementary materials:

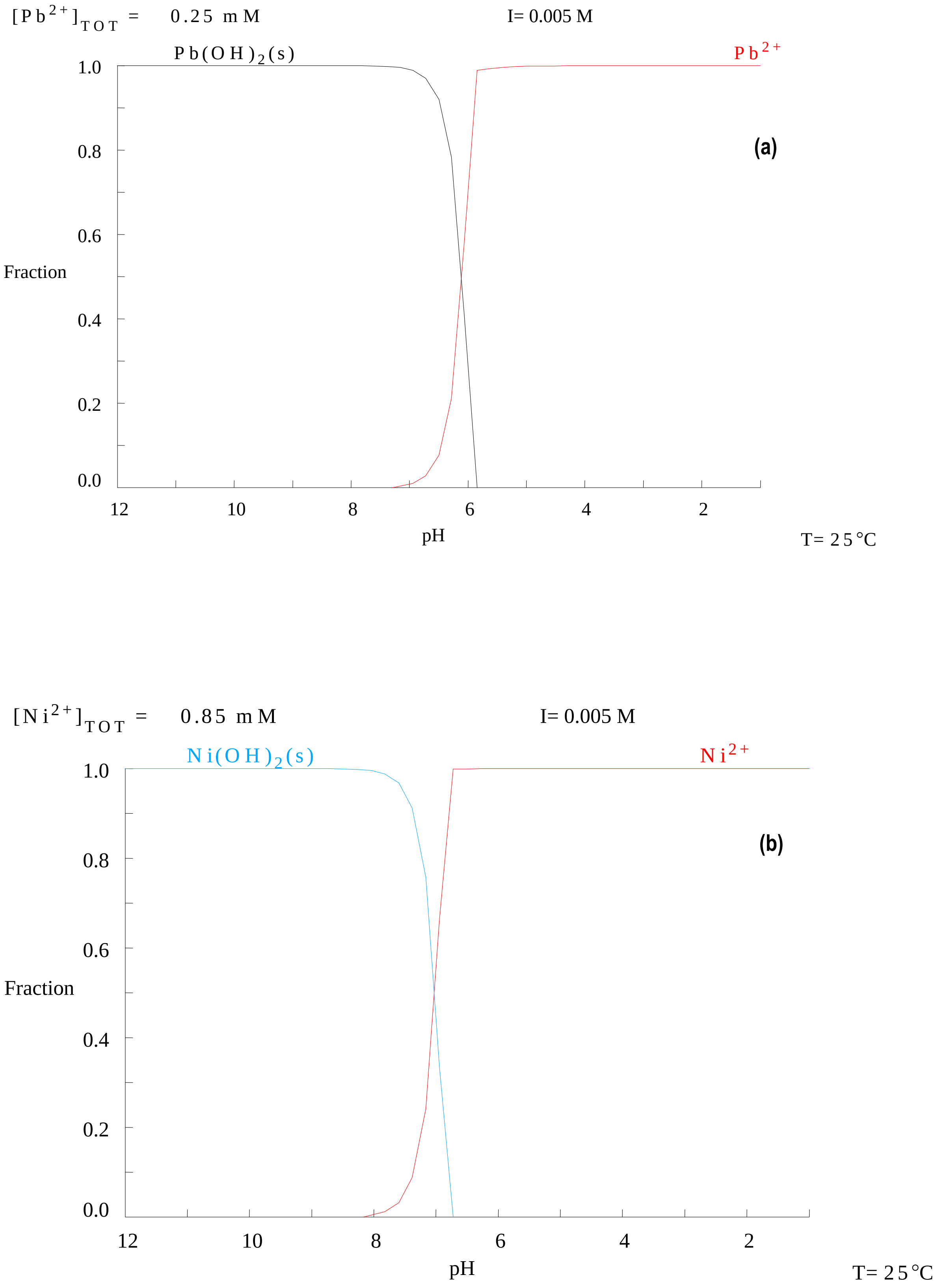

Figure S1. Metal species as a function of pH.

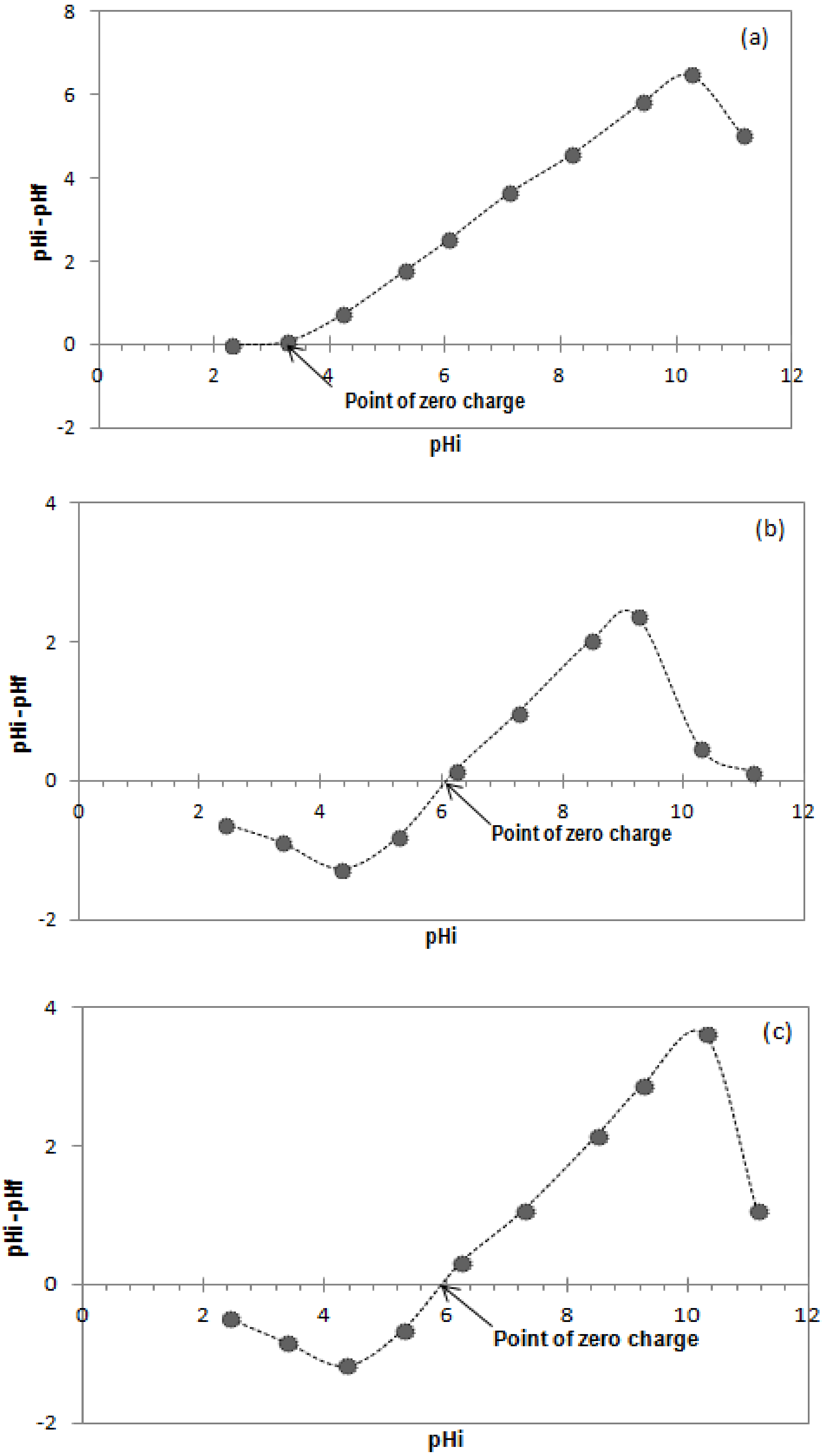

Figure S2. Points of zero charge of different sorbents. (a) HM-D2EHPA; (b) CA; (c) CAM-D2EHPA

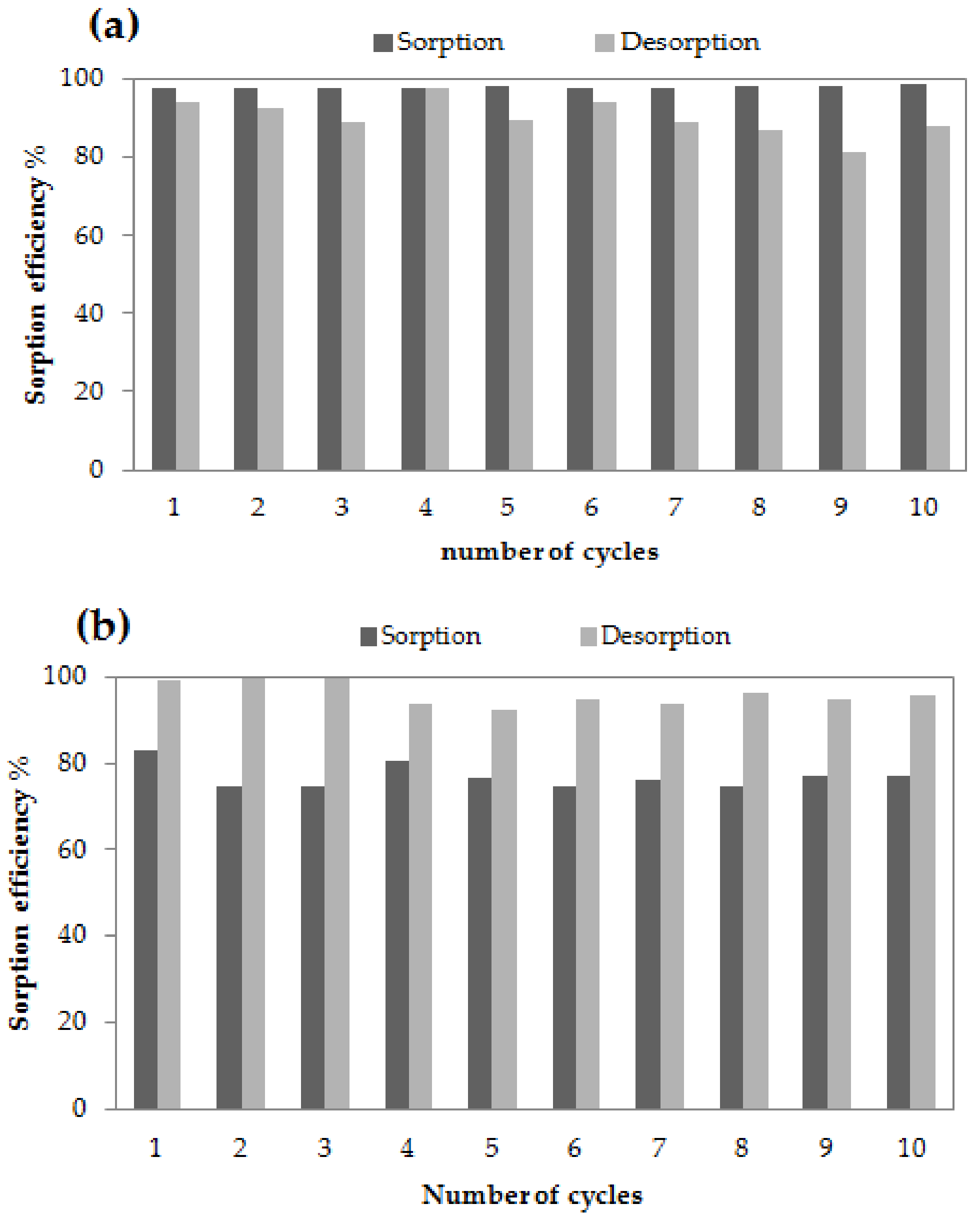

Figure S3. Sorption and desorption efficiency of lead (a) and nickel (b) by CAM-D2EHPA material. (T: 20 °C; sorbent dosage, SD: 10 g L^{-1}; agitation speed: 180 rpm; contact time: 3 h; C_0: 50 mg L^{-1}; C (HNO_3): 0.5 M; time of desorption: 30 min).

Table S1. Langmuir, Freundlich, and Sips constants of CA material.

	Langmuir				Freundlich			Sips			
Metal	q_{exp} (mg g^{-1})	q_{max} (mg g^{-1})	k_L (L mg^{-1})	r^2	k_F ($mg^{1-1/n}$ g^{-1} $L^{1/n}$)	n_F	r^2	q_{max} (mg g-1)	k_S (L mg^{-1})	n_S	r^2
Pb(II)	197.7	203.0	0.023	0.989	27.00	3.22	0.956	217.55	0.036	1.20	0.991
Ni(II)	47.6	51.32	0.020	0.995	9.31	3.97	0.956	53.83	0.034	1.19	0.997

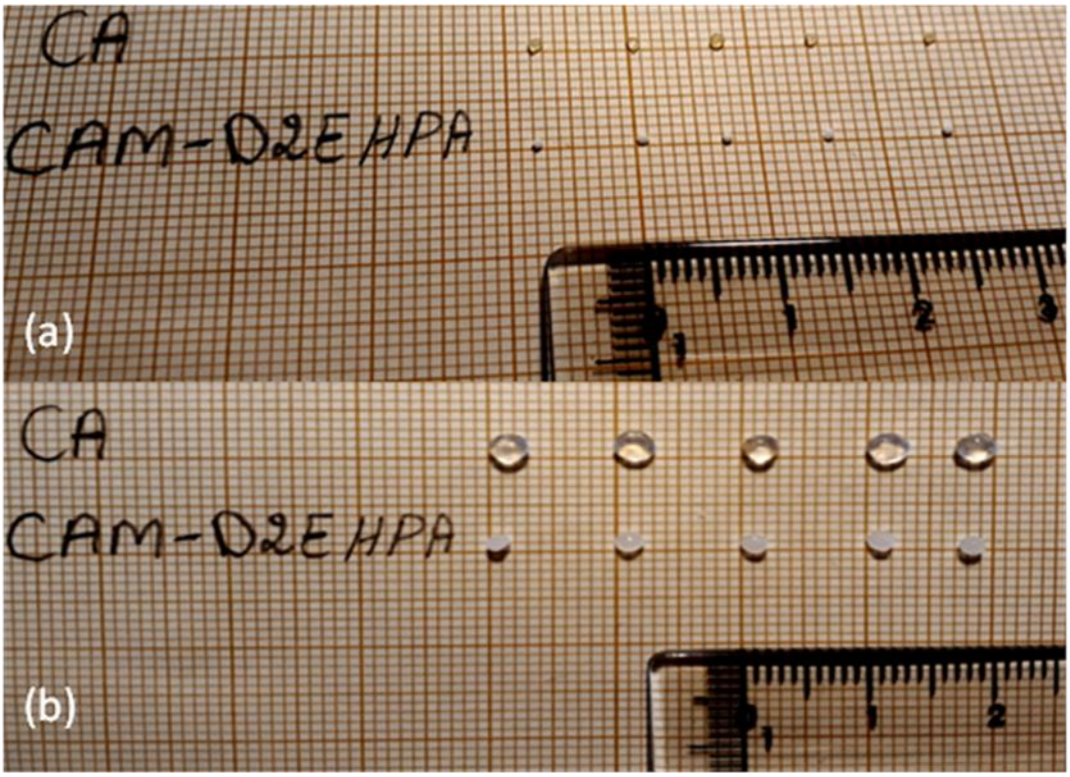

Figure S4. Images of calcium alginate beads, and CAM-D2EHPA hybrid beads; a) before adsorption, b) after adsorption
